# Targeting the NF-κB Pathway in Cancer: Mechanisms, Resistance, and Therapeutic Potential Across Tumor Types

**DOI:** 10.3390/ph18111764

**Published:** 2025-11-20

**Authors:** Kara Lukas, Jessica Nguyen, Clare Necas, Kushal Dave, Vishwanath Venketaraman

**Affiliations:** College of Osteopathic Medicine of the Pacific, Western University of Health Sciences, Pomona, CA 91766, USA; kara.lukas@westernu.edu (K.L.);

**Keywords:** NF-κB, drug discovery, cancer, tumor, metastasis

## Abstract

Cancer remains a leading cause of death, and current therapeutic options designed to slow the progression of cancer or eradicate cancer cells are often limited by drug resistance, inefficacy, or adverse effects. The Nuclear Factor Kappa B (NF-κB) pathway is a central regulator of inflammation and immune responses, and its dysregulation contributes to cancer development and progression. This review provides an overview of the role of the NF-κB pathway in tumor development and progression and discusses the potential of targeting specific modulators of the pathway for cancer drug discovery, specifically cancers that have the highest prevalence, such as breast, colorectal, lung, melanoma, and prostate cancers. While NF-κB inhibitors show promise, particularly in hematologic malignancies, challenges remain in translating these findings to solid tumors due to pathway complexity and its essential role in normal immunity.

## 1. Introduction

**Figure 1:**
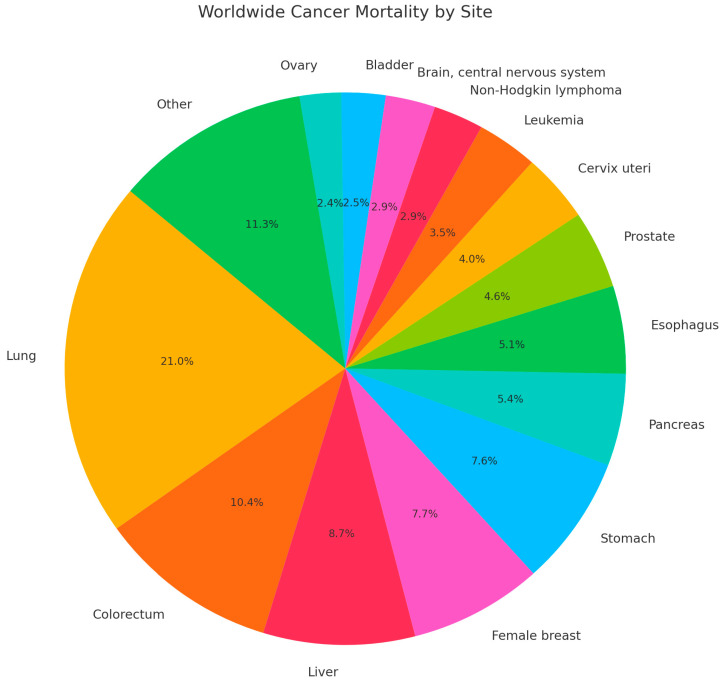
Worldwide Cancer Mortality by site in 2022.

Cancer mortality rates have continued to decline due to health education, smoking reductions, earlier detection for some cancers, and improved treatment [1]. However, despite the advancements in treatment and improved cancer screening methods, there continues to be a high worldwide mortality rate, as seen in Figure 1 with the most recent global data from 2022 [2]. Given the persistent global burden of cancer, continued exploration of the molecular mechanisms underlying tumor development and resistance to therapy remains essential. One key pathway implicated in these processes is the Nuclear Factor Kappa B (NF-κB) pathway, which will be explored in this review.

The NF-κB signaling pathway is a well-known pathway involved in inflammatory and immune responses, where disruption of the pathway can result in various complications such as inflammatory diseases, cardiovascular disease, neurodegenerative disorders, and cancers [3]. Looking more specifically at the association between NF-κB and cancer, activation of the pathway plays a significant role in tumor development and progression through mediation of cell survival, differentiation, and proliferation by regulating the expression of numerous biologically significant genes, including apoptosis regulators, stress-response genes, cytokines, chemokines, growth factors, and their receptors [4]. This review discusses the potential for targeting the NF-κB pathway as a mechanism to address tumor progression and chemotherapy resistance by examining its involvement in these processes as a desirable target for cancer therapy.

## 2. Methods

We conducted a literature search in PubMed and Google Scholar to identify relevant studies on the NF-κB signaling pathway and its role in cancer. Search terms included “NF-κB pathway in cancer”, “NF-κB pathway”, “NF-κB and skin cancer”, “NF-κB and breast cancer”, etc. Additional keywords were combined as appropriate to refine the search for specific cancer types. The reference lists of selected articles were also reviewed to identify further relevant articles. We prioritized articles published in the last five years.

## 3. Overview of NF-κB Pathway

There are five subunits of the NF-κB family: NF-κB1 (p105/p50), NF-κB2 (p100/p52), RELA (p65), cREL, and RELB, all of which can create a multitude of dimeric combinations with each other. These NF-κB dimers are typically sequestered in the cytoplasm and kept inactive by IκBs, a group of inhibitory proteins. However, this inhibition is reversed upon activation of inhibitory-kappa B kinase (IKK), an enzyme that phosphorylates IκBs and targets them for degradation. This degradation releases the NF-κB dimers, allowing them to translocate into the nucleus and activate their target genes. As part of this negative feedback loop, IκBs are then resynthesized to terminate the NF-κB response by binding NF-κB in the nucleus and exporting it back to the cytoplasm [5].

### 3.1. Canonical and Non-Canonical Pathways

**Figure 2:**
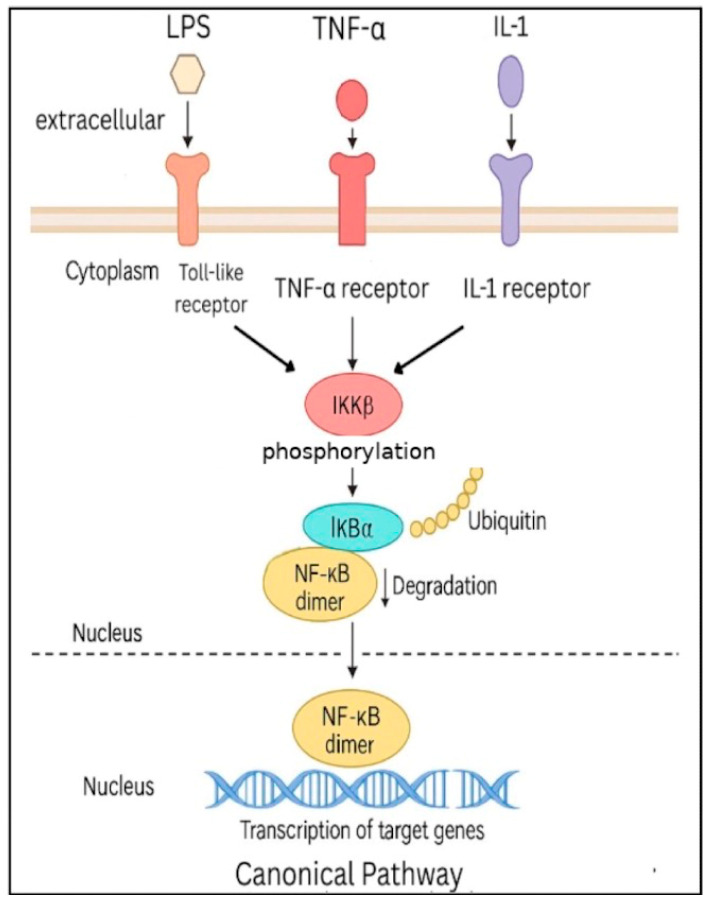
Canonical NF-κB Pathway. NF-κB dimers are released from IκB inhibitors upon activation of IKKB, allowing nuclear translocation and transcription of target genes. The canonical pathway also interacts with other signaling networks, including STAT3, PI3K/MAPK, and inflammasome pathways, which can influence NF-κB activity.

**Figure 3:**
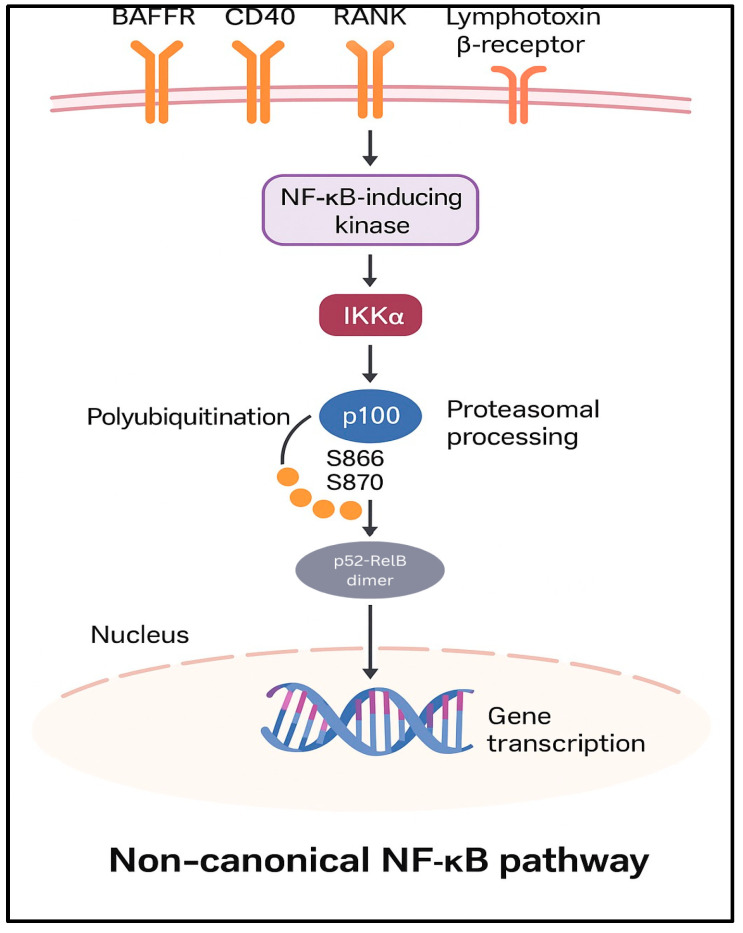
Non-canonical NF-κB Pathway. Activation of receptors such as BAFFR, CD40, RANK, or Lymphotoxin ß receptors leads to IKK∂-mediated processing of p100 to p52, allowing nuclear translocation of p52/RelB dimers. The non-canonical pathway can similarly interface with STAT3, PI3K/AKT, MAPK, and inflammasome pathways, influencing NF-κB activity.

The regulation of NF-κB activity has been described via two pathways: the canonical (classical) pathway and the non-canonical (alternative) pathway, as seen in Figure 2 and Figure 3, respectively. In the canonical NF-κB signaling pathway, stimuli such as lipopolysaccharides (LPS), tumor necrosis factor-alpha (TNF-α), and interleukin-1 (IL-1) activate their respective receptors—Toll-like receptors, tumor necrosis factor receptors, and interleukin-1 receptors (IL-1R) [6]. These signals are transmitted through various adaptor proteins and kinases, ultimately leading to the activation of IKKβ, a key catalytic subunit within the IKK complex; whereas, activated IKKβ phosphorylates IκBα at serine residues 32 and 36, marking it for polyubiquitination and subsequent proteasomal degradation, which releases NF-κB dimers, allowing dimers to enter the nucleus to initiate transcription of target genes [7].

In the non-canonical NF-κB pathway, stimulation of receptors such as BAFFR, CD40, RANK, or the lymphotoxin β-receptor initiates a signaling cascade involving the NF-κB-inducing kinase; activation of IKKα subsequently phosphorylates the p100 protein at serine residues 866 and 870 and marks p100 for polyubiquitination and proteasomal processing, resulting in its conversion to p52 [5]. The resulting p52-RelB heterodimers translocate to the nucleus, where they regulate the transcription of specific target genes [8].

### 3.2. Role of NF-κB in Different Cellular Pathways

The NF-κB pathway has been associated with many cellular processes, in particular, inflammation, where the NF-κB p50 and p65 subunits have shown the ability to bind to NF-κB site on the IL-6 gene, a gene known to produce the inflammatory cytokine IL-6 [9,10]. Additionally, nobelitin, a polymethoxyflavonoid found in citrus fruits, has been shown to promote anti-inflammatory effects in muscle cells through inhibition of the NF-κB pathway, suggesting the pathway, when active, plays a role in inflammatory processes [11].

Immune response has also been shown to be affected by the NF-κB pathway, where, for example, the NF-κB subunit RelB is essential for the formation of the thymic medullary epithelial cells and dendritic cells, both of which are critical components of the immune system [12]. Furthermore, NF-κB activity is essential at two key stages of B cell development: first, to support the survival of pre-B and immature B cells, and later, to promote both the survival and maturation necessary for the formation of follicular mature B cells [13].

Furthermore, the pathway has been shown to play a role in cell cycle progression and inhibition of apoptosis as well; for example, loss of NF-κB1 subunit enhances apoptosis in quiescent B cells and Rel and NF-κB1 are essential for mitogen-activated B-cell survival [14]. Another study showed how NF-κB activity is critical for the upregulation of cyclin D1, subsequent hyperphosphorylation of retinoblastoma protein, and the promotion of G1-to-S phase cell cycle progression [15].

The NF-κB pathway is a central regulatory hub that orchestrates diverse cellular processes including inflammation, immune responses, cell survival, and apoptosis, often interacting with other key pathways such as STAT3, PI3K/AKT, and MAPK, highlighting its critical role in both normal physiology and disease pathogenesis, and ultimately serving as a key integrator of cellular signaling [11,12,14]. In the context of cancer, these same functions become co-opted to drive tumor development and progression, making NF-κB a critical focus of the following discussion.

### 3.3. Role of NF-κB in Cancer Progression

The NF-κB pathway is a known pathway involved in activating inflammatory responses and promoting oncogenesis, acting as a driver of chemoresistance, which interferes with the efficacy of current chemotherapy treatments [16]. NF-κB pathway inhibitors have shown the greatest clinical success in hematologic malignancies, particularly multiple myeloma and Waldenström’s macroglobulinemia, as demonstrated in multiple phase III clinical trials [17,18,19]. In solid tumors, effective translation remains challenging due to pathway complexity and compensatory mechanisms, motivating ongoing research into combination therapies and targeted approaches [20]. Depending on the cancer type, the NF-κB pathway can be targeted with different key molecules, as depicted in Table 1. The following subsections provide a cancer-type-specific overview of NF-κB activation, key molecular mediators, and potential therapeutic strategies, highlighting both preclinical findings and challenges in clinical translation.

## 4. NF-κB in Cancer

### 4.1. NF-κB and Breast Cancer

Breast cancer is the most common cancer diagnosed in females in 2025 and is the leading cause of cancer death among women younger than 50 years [1]. One study examined TRIM32, a member of the tripartite motif (TRIM) family of proteins, overexpressed in human breast cancers and mediates cisplatin resistance through activation of the NF-κB signaling pathway; subsequently, it was found that inhibition of the NF-κB pathway decreased the effects of TRIM32 and ultimately downregulated cell viability [21]. Another study showed how the NF-κB pathway plays a role in enhancing stemness and radioresistance in breast cancer stem cells (BCSCs) by regulating MIR155HG, a long non-coding RNA MIR155 host gene (MIR155HG) located on human chromosome 21, via transcriptionally activating the Wnt pathway [22]. The Wnt signaling pathway plays a crucial regulatory role in maintaining tissue homeostasis within the tumor microenvironment of breast cancer cells and acts as a major driver of carcinogenesis [23]. Therefore, targeting NF-κB to affect MIR155HG may increase cancer stem cells’ vulnerability to radiation-induced cell death, potentially enhancing therapeutic efficacy.

Ferroptosis has been shown to play a major role in tumor suppression [24]. Paclitaxel (PTX) is a common chemotherapeutic agent and has been shown to induce ferroptosis in colorectal cancer [25]. Similarly, in triple-negative breast cancer (TNBC), RSL3 induces ferroptosis through direct inhibition of glutathione peroxidase 4 [26]. One study explored the synergistic effects of PTX and RSL3 in TNBC and found that RSL3 was able to induce ferroptosis by activating the NF-κB pathway, which allowed for increased chemosensitivity of TNBC to PTX [27].

Antimicrobial peptides have also been explored for their potential anticancer effects. One study demonstrated that Moricin, an antimicrobial peptide, triggered caspase-dependent cell death in TNBC cells by downregulating the expression of key proteins, including Notch-1 and NF-κB proteins. This suppression activated apoptotic pathways, leading to increased cell death as well as inhibition of cancer cell proliferation, specifically in the MDA-MB-231 cells derived from metastatic TNBC tumors [28].

Collectively, these studies highlight how NF-κB activation in breast cancer promotes chemoresistance, stemness, and survival signaling. Targeting NF-κB, either directly or via upstream modulators such as TRIM32 or MIR155HG, may sensitize tumors to existing therapies, although careful consideration is needed given the pathway’s essential role in normal immune function.

### 4.2. NF-κB and Colorectal Cancer

Colorectal cancer remains the second-most common cause of cancer death in men and women combined, with 52,967 reported deaths in 2022, even though overall mortality rates have been decreasing [1]. 5-fluorouracil (5-FU) is the current first-line therapy for patients with advanced colorectal cancer; however, due to its high drug resistance and toxicity, creating a new treatment with low toxicity and less potential to develop drug resistance has become the focus of current CRC research [29]. One emerging therapeutic strategy is modulation of nucleolar function. Nucleolar dysfunction is now recognized as a hallmark of cancer, promoting tumor growth by supporting the protein synthesis needed for rapid cell proliferation and disrupting key nucleolar pathways that regulate cell growth and death [30].

First-line treatment for patients with microsatellite stable, RAS wild-type, and left-sided metastatic colorectal cancer consists of anti-EGFR (epidermal growth factor receptor) plus chemotherapy [31]. It has been shown that persistent activation of the NF-κB pathway decreases sensitivity to anti-EGFR monoclonal antibodies and the continued activation of the pathway results in an inflammatory tumor microenvironment, which reduces the effectiveness of anti-EGFR monoclonal antibody treatment. However, colorectal cancer cells exhibiting oscillatory NF-κB activity responded better to anti-EGFR therapy, suggesting that oscillations may dampen pro-inflammatory signaling and improve treatment outcomes. The findings indicate that the pattern of NF-κB activity, whether sustained or oscillatory, significantly influences the response to anti-EGFR monoclonal antibodies in CRC. Tracking NF-κB oscillation profiles may serve as a predictive biomarker for treatment efficacy, aiding in the development of personalized therapeutic approaches [32].

The NF-κB pathway is a known pathway involved in activating inflammatory responses and promoting oncogenesis, which interferes with the efficacy of current chemotherapy treatments and contributes to chemoresistance [16]. Recent studies have also emphasized the role of chronic inflammation triggered by microbial infections, such as those from *Helicobacter pylori* and *Fusobacterium nucleatum*, in contributing to tumorigenesis through the activation of the NF-κB pathway [33]. Consequently, many studies are being conducted to examine the effects of inhibiting the NF-κB pathway to improve colorectal cancer treatment and reduce recurrence rates. Additional studies have looked at how the pathway may be targeted in patients with autoimmune conditions like Type 1 diabetes. One study found the p65 subunit is essential in driving tumor growth and shaping the immune microenvironment in CRC, especially in the setting of T1D. The study further demonstrated that knockdown of the p65 subunit in tumor cells reduced tumor progression and decreased immune evasion mechanism to potentiate the anti-tumor response [34]. These findings indicate that inhibiting the NF-κB pathway could enhance cancer immunotherapy effectiveness, particularly in patients with autoimmune conditions such as T1D.

MUC13 is a transmembrane mucin glycoprotein that is overproduced in colon cancer tissue, which upregulates BCL-XL expression via activation of the NF-κB pathway and protects cancer cells from cell death [35]. One study demonstrated that MUC13 is required in colonic cells for activation of NF-κB and found that silencing MUC13 significantly reduced NF-κB activation and subsequently sensitized colorectal cancer cells to death [29]. Similarly, another study examined diHEP-DPA, a docosahexaenoic acid derivative (DiHEP), and demonstrated that combining it with chemotherapy effectively suppresses the infiltration of tumor-associated macrophages via inhibition of the NF-κB pathway. This combination enhances the effects of chemotherapy and prevents chemoresistance, highlighting diHEP-DPA’s potential as both a therapeutic agent and a prognostic marker [36]. One preclinical study investigated the drug repurposing of enalapril, a common antihypertensive angiotensin-converting enzyme (ACE) inhibitor, given that ACE converts angiotensin I to angiotensin II and plays a role in activating NF-κB. Using CRC cell lines and animal models, it was found that the combined use of enalapril and 5-FU greatly suppressed the NF-κB/STAT3 pathway, which restored chemosensitivity and potentiated the antitumor effects of 5-FU [37]. Further studies are thus needed to determine the safety and efficacy in humans.

Additionally, herbal remedies have been explored for their effect on colorectal cancer progression. Banxia Xiexin Decoction (BXD) is a Chinese herbal formula often given to patients to manage gastrointestinal disorders. One study explored its effects in colon cancer and found that the proliferation of colon cancer cells was significantly reduced following BXD intervention. The mRNA and protein levels of PARG, PARP1, and NF-κB p65 were downregulated by BXD, suggesting that BXD may suppress the malignant characteristics of colon cancer cells by modulating the PARG/PARP1/NF-κB signaling pathway [38]. Additionally, PAMAM dendrimers conjugated with gallic acid have been shown to inhibit NF-κB activation and promote apoptosis in colon cancer cells, offering another approach for colorectal cancer treatment [39].

Overall, these studies highlight the multifaceted role of NF-κB in colorectal cancer progression, chemoresistance, and modulation of the tumor immune microenvironment. Targeting NF-κB signaling, either directly or through modulators such as MUC13 or the NF-κB/STAT3 axis, offers potential therapeutic benefit. As most evidence remains preclinical, further studies are required to determine safety, efficacy, and translational potential.

### 4.3. NF-κB and Lung Cancer

Lung cancer remains one of the leading causes of cancer-related mortality worldwide, with non-small cell lung cancer (NSCLC) accounting for the majority of cases [1]. The NF-κB pathway plays a critical role in lung cancer progression, influencing cell proliferation, survival, metastasis, and therapeutic resistance. TRIM32, which is also implicated in breast cancer, is overexpressed in NSCLC, inducing cell proliferation, colony formation, and invasion, and mediating cisplatin chemotherapy resistance through activation of the NF-κB/Bcl-2 signaling pathway. Inhibition of NF-κB attenuates TRIM32-induced Bcl-2 upregulation and suggests its potential as a target to reduce resistance to cisplatin chemotherapy in NSCLC [40].

Looking specifically at lung cancer metastasis to the brain, pemetrexed is a first-line treatment; however, the effectiveness of the treatment is limited by drug resistance. It was found that CD146 plays a large role in pemetrexed resistance by inhibiting apoptosis via upregulation of the NF-κB pathway, and inhibition of the pathway significantly increased the sensitivity of brain metastatic cells to pemetrexed while reducing CD146′s effect on pemetrexed resistance [41].

Interestingly, NF-κB activation can also have context-dependent effects. DDX24, a splicing factor that is significantly elevated in lung tissue, was found to promote autophagy and suppress lung cancer growth when deleted. This occurs through the production of a longer IKBKG isoform, which activates NF-κB and increases the transcription of the BECN1 gene [23]. Targeting DDX24 may therefore offer a promising therapeutic approach in the treatment of lung cancer.

Immune checkpoint inhibitors (ICIs) are a mainstay of cancer treatment, but resistance remains a challenge [42]. One study revealed that in patients with NSCLC, ICI non-responders had a higher presence of CD4+ regulatory T cells, resident memory T cells, and TH17 cells, in contrast to the diverse, activated CD8+ T cells observed in responders. Additionally, tumor cells in non-responders often displayed increased transcriptional activity in the NF-κB and STAT3 pathways, indicating a potential intrinsic resistance to ICI therapy [43]. Thus, suppressing the NF-κB pathway may prove to be beneficial in those treated with ICIs.

These studies demonstrate the complex role of NF-κB in lung cancer and suggest that modulating NF-κB activity through targets such as TRIM32, CD146, and DDX24 is a potential therapeutic strategy.

### 4.4. NF-κB and Melanoma

Melanoma is a highly invasive skin cancer with a rapidly increasing worldwide incidence [44,45]. The NF-κB signaling pathway is constitutively active in melanoma cells and plays a key role in regulating genes involved in various processes, including cell proliferation and survival, inflammation, invasion, angiogenesis, and apoptosis [46]. Additionally, activation of the Rela/NF-κB pathway in microglia facilitates melanoma brain metastasis. Inhibiting this pathway reprograms microglia into a proinflammatory state, strengthening antitumor immunity and reducing the metastatic burden in the brain [47]. Consequently, the regulation of NF-κB pathway activity has been targeted in several promising therapeutic strategies aimed at benefiting patients with melanoma.

NF-κB inhibitors have long been discussed as having potentially significant anti-tumor effects in melanoma tumors [48]. One preclinical study using melanoma cancer cell lines WM-266-4 and B16F10 recently showed that serotonin type-3 (5-HT3) receptor antagonists, such as tropisetron and ondansetron, demonstrated a selective concentration-dependent toxic effect on melanoma cells through inhibition of NF-κB localization in the nuclei [49]. Similarly, another study identified secreted phosphoprotein 1 (SPP1) as a driver of melanoma that can be regulated by bromodomain and extra-terminal domain (BET) inhibitors, which target the noncanonical NF-κB/SPP1 pathway through inhibition of NF-κB2. Inhibition of NF-κB2 leads to decreased SPP1 expression, thereby suppressing melanoma growth and progression. These findings suggest that BET inhibitors may have therapeutic potential in the treatment of melanoma [44]. Additionally, it was recently determined that 17-aminogeldanamycin can effectively inhibit NF-κB activity in melanoma cell lines by targeting heat shock protein 90, which regulates the p65/NF-κB signaling pathway. This inhibition leads to reduced phosphorylation of the p65 subunit at Ser536, which is an important marker of NF-κB activation, and subsequently decreases the expression of NF-κB target genes, including IL-8 and VEGF. These changes contribute to the induction of apoptosis in melanoma cells and have implications for inhibiting melanoma metastasis and angiogenesis, highlighting the potential of 17-aminogeldanamycin as a single agent or adjuvant therapy in the treatment of patients with melanoma [46]

Another recent preclinical study explored the potential of regulating the NF-κB pathway through microalgal carotenoids, including astaxanthin, fucoxanthin, and zeaxanthin, as a therapeutic approach for melanoma. These carotenoids were shown to inhibit NF-κB activation, which resulted in decreased melanoma cell proliferation, migration, and invasion, as well as increased apoptosis. While this highlights their potential as adjuvants in melanoma therapy, factors such as bioavailability and pharmacokinetics remain important considerations for clinical application [50]. Various combinations of therapeutic agents have also been explored in the treatment of patients with melanoma. One study focused on the effect of combined BET and MCL 1 inhibitors and determined that this combination induces apoptosis in melanoma cells by downregulating NF-κB-regulated anti-apoptotic proteins, including BCL2A1 and XIAP, while upregulating pro-apoptotic proteins, such as BIM and NOXA. The study additionally indicated that BCL2 family inhibitors, when added to BET and MCL1 inhibition, have the potential to enhance therapeutic efficacy and help overcome treatment resistance [51]. Another study determined that the combination of celecoxib and trametinib increased total NF-κB protein expression while simultaneously inhibiting NF-κB activation, leading to an antiproliferative and proapoptotic therapeutic effect in melanoma treatment [52].

Together, these studies highlight the role of NF-κB in melanoma progression, metastasis, and therapy resistance. Modulating NF-κB activity through inhibitors, natural compounds, or combination strategies is a promising therapeutic avenue. Given that most evidence remains preclinical, further translational studies are needed to evaluate clinical efficacy and safety.

### 4.5. NF-κB and Prostate Cancer

Prostate cancer affects men ages 45 to 60, with around 1 in 8 men being diagnosed with prostate cancer in their lifetime. After lung cancer, prostate cancer is the second-leading cause of cancer-related deaths in men, with around 1 in 44 men dying of prostate cancer [53,54]. While 1 in 8 men can be diagnosed with prostate cancer, the risks vary based on various factors such as genetics. Men with a close family relative with a diagnosis of prostate cancer have a 50% risk of developing prostate cancer compared to those that do not have a relative with prostate cancer [53]. The current treatments for prostate cancer include active surveillance, radical prostatectomy, external beam radiation, brachytherapy, cryotherapy, hormone therapy, and chemotherapy with various potential adverse effects. These treatments are dependent on localization of the cancer and the advancement [53].

In castrate-resistant prostate cancer (CRPC), the NF-κB pathway is frequently activated, which contributes to tumor progression and resistance to treatment therapies [55]. Androgen deprivation therapy (leuprolide, goserelin, triptorelin) and antiandrogen therapies like enzalutamide (ENZ) are used to treat castrate-resistant metastatic diseases [53,56]. However, after initial treatment and response to ADT, patients can develop resistance to androgen therapies, leading to CRPC [57]. Preclinical studies indicate that inhibition of IκKβ and BCL-2 can prevent the emergence of ENZ resistance in in vivo models and may overcome therapy resistance [56]. One study found that Dimethylamino Parthenolide (DMAPT), a parthenolide analogue, inhibited the NF-κB canonical pathway by preventing the p65 subunit on IκKβ from binding to DNA, leading to apoptosis. In combination with ENZ, DMAPT reduced cell survival by 73–77% in vitro and decreased androgen receptor variant-7, suggesting potential restoration of ADT responsiveness in CRPC [58].

Similarly, inhibition of the IKK complex with BMS345541, a selective small-molecule inhibitor of the IKK complex, and bortezomib effectively reduced the expression of androgen receptor variants and restored sensitivity to therapy in preclinical models [55]. Another recent preclinical study demonstrated that artesunate (AS) can sensitize CRPC cells to androgen receptor antagonists, such as bicalutamide (Bic). When AS is used in combination with Bic, NF-κB signaling is inhibited at multiple key points, including a reduction in phosphorylation of the p65 subunit. This inhibition also results in the restoration of the sensitivity of prostate cancer cells to treatment [59]. In addition, the long noncoding RNA DRAIC interacts with IKK subunits to prevent their interaction with each other, IκBα phosphorylation, and NF-κB activation [60].

Beyond direct modulation of CRPC therapies, NF-κB signaling may also influence prostate cancer progression via systemic factors, including the gut microbiome. A potential biomarker for the progression of prostate cancer is Proteobacteria. One study found antibiotic-induced gut dysbiosis, marked by an increase in Proteobacteria, heightened gut permeability and intratumoral LPS levels, driving prostate cancer development in mice through the NF-κB-IL6-STAT3 pathway [61].

In addition to experimental therapies, commonly used drugs such as aspirin may exert anti-cancer effects by targeting NF-κB signaling. NF-κB regulates the expression of cyclooxygenase (COX) which is important in cell growth in prostate cancer because it drives prostaglandin biosynthesis. Aspirin (acetylsalicylic acid), a COX inhibitor, can inhibit the NF-κB pathway. When looking at the DU-145 prostate cancer cell line, an androgen-independent prostate cancer cell line, aspirin inhibited both DNA and protein synthesis [62]. A population-based cohort study conducted in Sweden followed adult male aspirin and NSAID users from when they were first given these medications until December 2012, first cancer diagnosis, or death, whichever occurred first. They found that longer durations of use (>5 years) of aspirin and other NSAIDs provided evidence that these drugs had a protective effect against prostate cancer [63]. However, there are varying results from several epidemiological studies, ranging from no benefits towards prostate cancer when taken after cancer diagnosis to aspirin use being inversely related to prostate cancer incidence and significantly inversely related to developing advanced prostate cancer [64,65]. Further exploration into how aspirin inhibits prostate cancer progression while minimizing the side effects of prolonged NSAID use is needed to support clinical translation.

Overall, these studies reveal the role of the NF-κB pathway in prostate cancer and highlight various ways that drugs, both experimental and repurposed, can target this pathway to improve patient outcomes.

### 4.6. NF-κB and Other Cancers

The NF-κB pathway has been implicated in the progression and therapy resistance of numerous cancers, making it a promising target for drug development across diverse tumor types. Drugs that inhibit NF-κB signaling, as well as natural compounds and repurposed agents, have shown potential in preclinical and clinical studies. In the following subsections, we explore recent findings in specific cancer types.

#### 4.6.1. Gastric Cancer

In gastric carcinoma, approximately 89% of noncardia gastric cancers are associated with *H. pylori* infection [66]. Uridine phosphorylase 1 (UPP1), an enzyme involved in pyrimidine metabolism, has been found to be elevated in *H. pylori*-infected gastric tissue within tumor cell populations [67]. Prior studies have shown that UPP1 expression is regulated by the NF-κB pathway, with *H. pylori* infection increasing UPP1 levels through the NF-κB pathway via P65 activation [68]. This relationship highlights the role of NF-κB signaling in mediating *H. pylori*-induced tumorigenic processes, suggesting targeting the NF-κB pathway may represent a promising therapeutic strategy for noncardia gastric cancer.

One study found that IL-8, an inflammatory cytokine, promotes chemoresistance to cisplatin in human gastric cancer via NF-κB activation and suggests targeting IL-8 to inhibit the downstream effects of NF-κB and enhance response to chemotherapy [69]. Similarly, Siva-1, an anti-apoptosis protein that is overexpressed in gastric cancer, was found to enhance the activity of NF-κB and is a contributor to multidrug resistance [70]. Additionally, another study found that Acetyl-keto-beta boswellic acid (AKBA) sensitized gastric cancer cells to cisplatin-induced apoptosis by modulating the p53 pathway. When combined, AKBA and cisplatin significantly increased p53 expression, reduced NF-κB levels, and promoted apoptosis in a dose-dependent manner, highlighting the potential for the use of AKBA as an adjuvant in the treatment of patients with gastric cancer [71].

#### 4.6.2. Nasopharyngeal Cancer

Activation of the NF-κB signaling pathway also plays a role in nasopharyngeal carcinoma (NPC). It is known that Epstein–Barr virus (EBV) infection results in the activation of STAT3 and NF-κB signal cascades in nasopharyngeal epithelial cells and facilitates NPC development [72]. One study demonstrated that inhibiting the NF-κB signaling can interrupt EBV latency in nasopharyngeal carcinoma cells by downregulating BamHI-A rightward transcripts (BARTs) expression and inducing lytic cell replication, suggesting that the NF-κB pathway can be a potential target for future treatment of EBV-associated carcinomas [73]. Furthermore, latent membrane protein 1 (LMP1), a tumorigenic transmembrane protein, is also upregulated by NF-κB activation with downstream activation of glucose transporter 1 transcription and promotes the growth of NPC cells as well as facilitates pathogenesis. Similarly, MiR-125b, a microRNA, is upregulated in NPC tissue and contributes to NPC development by activating the NF-κB pathway and creating downstream downregulation of A20, a tumor suppressor. This suggests that targeting the miR-125b/A20/NF-κB signaling axis may serve as a novel therapeutic approach to treating NPC [74].

#### 4.6.3. Bladder Cancer

In bladder cancer, inhibition of the NF-κB pathway has been shown to result in the overexpression of survivin, a molecular marker associated with poor clinical outcomes and malignant progression. Interestingly, Cui [75] reported that YM-155, a molecule that selectively suppresses survivin expression and suppresses bladder cancer growth, is enhanced by NF-κB activation [75]. Additionally, TRIM29 is overexpressed in bladder cancer tissues, where it promotes tumor progression by activating the protein kinase C (PKC) and NF-κB pathway, thereby inhibiting cancer cell apoptosis. Treatment with the PKC inhibitor, staurosporine, was shown to block the pro-tumorigenic effects of TRIM29 mediated by NF-κB activation [76].

Moreover, the activation of the unfolded protein response (UPR), which is regulated by the NFKB-miR-29b/c axis, is critical in promoting tumor aggressiveness and disease progression in bladder cancer. This regulatory network highlights potential therapeutic targets and prognostic markers for managing the disease. Notably, the miR-29 family of miRNAs inhibits UPR-driven tumor aggressiveness, contributing to improved survival outcomes in bladder cancer [77].

#### 4.6.4. Osteosarcoma, Cervical, and Bone Marrow Cancers

In osteosarcoma, the combination of curcumin and methotrexate showed a trend of reduced expression of NF-κB and matrix metalloproteinases, indicating potential synergy for anti-tumor effects [78].

In cervical cancer, one study found that Ovatodiolide, a plant-derived macrocyclic diterpenoid, decreases NF-κB expression and contributes to anticancer effects through induction of apoptosis and cell cycle arrest, highlighting its potential as a therapeutic agent [79].

In myelofibrosis and other bone marrow malignancies, the curaxin-derived drug CBL0137 targets the non-canonical NF-κB signaling pathway and inhibits its downstream effects, including TNF receptor signaling and NIK expression. This finding suggests that modulating the NF-κB pathway through this mechanism could improve treatment outcomes in myeloproliferative neoplasms, particularly those that are driven by JAK2 mutations [80].

#### 4.6.5. Multiple Cancers and Emerging Therapies

Several plant-derived and repurposed drugs demonstrate NF-κB modulation across multiple cancer types. One study found that plant-derived anticancer agents, such as Alkaloids and polyphenols, can serve as anti-cancer agents by disrupting cellular processes and deactivating key signaling pathways, including the NF-κB pathway in liver and breast cancer cells [81]. Another study found that Peruvoside, a novel cardiac glycoside, modulates the NF-κB signaling pathway and contributes to its anti-cancer effects in breast, lung, and liver cancer cells [82].

Additionally, NF-κB inhibitors are being evaluated in combination with other therapeutics for solid tumors. For instance, one phase I study found that erlotinib, an EGFR inhibitor, in combination with ixazomib, an NF-κB inhibitor, was well-tolerated and demonstrated a promising preliminary antitumor activity in patients with advanced sarcoma [83].

#### 4.6.6. Summary

Collectively, these studies demonstrate the potential benefits of NF-κB inhibition in a wide range of cancers. Both experimental and repurposed drugs, as well as natural compounds, have demonstrated the ability to inhibit NF-κB activity, sensitize tumors to standard therapies, and reduce tumor growth in preclinical and early clinical settings. Targeting NF-κB directly or through upstream regulators thus represents a promising strategy for developing broad-spectrum anti-cancer therapies. Further research is needed to optimize these interventions, understand cancer type-specific mechanisms, and translate these findings into safe and effective clinical treatments.

## 5. Chemoresistance

While there are many available pharmaceutical options for cancer treatment, one challenge that continues to hinder the success of cancer treatment is the chemoresistance of drugs. In particular, there are clear examples of how NF-κB activation mediates acquired drug resistance, as seen in a recent study that demonstrated upregulation of NF-κB signaling as a key mechanism underlying acquired resistance to poly-adenosine ribose polymerase (PARP) inhibition, a class of anti-cancer drugs with proven activity in BRCA mutant cancers, while showing significant results that co-treating cancer treatment with an NF-κB inhibitor may reverse the acquired resistance [84]. Another study examined TNF-related apoptosis-inducing ligand (TRAIL) as a promising anticancer agent that has a similar limitation of acquired drug resistance and found that combinatory treatment of NF-κB inhibitors and TRAIL was able to reverse resistance and reduce tumor growth [85]. Looking specifically at the acquired drug resistance of EGFR tyrosine kinase inhibitors (TKIs) in NSCLC, it has been shown that inhibition of NF-κB is sufficient to reduce the viability of cancer cells that have adapted to EGFR TKIs [86].

There is an abundance of evidence demonstrating that activation of the NF-κB pathway is involved in chemotherapy resistance. Therefore, targeting the pathway is a potential therapeutic option for combating chemotherapy resistance.

## 6. Strategies for Targeting the NF-κB Pathway to Improve Therapeutic Outcomes

Researchers are interested in studying how targeting the NF-κB pathway can improve cancer therapy options, whether it is through inhibiting the pathway to mediate other inflammatory pathways, repurposing pharmaceutical options that are already available on the market, combining therapies to enhance the efficacy of certain drugs, or exploring alternative plant-based therapies. Because NF-κB is essential for normal immune function, broad inhibition carries significant toxicity risks; therefore, current strategies emphasize selective targeting of dysregulated NF-κB subunits or context-specific modulation within tumor cells versus immune cells.

### NF-κB and Modulation of the Tumor Microenvironment

The NF-κB pathway plays an important role in shaping the tumor microenvironment (TME), which broadly includes many different cell types, including immune cells, cancer-associated fibroblasts (CAF), tumor associated macrophages (TAM), endothelial cells, cytokines, and growth factors, with each cell type playing an individual role in tumor suppression or survival, providing strong evidence for targeting the NF-κB pathway as a means for cancer therapy. For example, CAFs not only provide nutrients and support blood vessel formation to help tumors grow, but also triggers inflammation, which attracts macrophages, helps form new blood vessels, and makes tumors grow faster. Blocking NF-κB signaling prevents these tumor-promoting effects from occurring and also prevents normal fibroblasts from becoming reprogrammed to behave like CAFs [87].

To highlight the newest developments in cancer research, researchers have created organoid cancer models with the incorporation of patient-derived tumor cells and fibroblasts to simulate the TME [88]. Specifically, enhanced research methods using 3D culture modeling, compared to a conventional 2D model, have been shown to most resemble the TME and are most predictive of human therapeutic efficacy. Likewise, researchers created NPC patient-derived xenografts to establish 3D culture models of NPC and mimicked the TME with co-culture of the organoids with human immune cells and CAF [89]. Utilizing transcriptomic profiling of patient-derived pancreatic tumor organoids, it was discovered that CK21, a novel triptolide analog, downregulates the NF-κB pathway and induces tumor cell apoptosis, suggesting a possible biomarker to target for pancreatic cancer treatment [90].

Through the use of co-cultured organoids and fibroblasts, it was found that CAF plays a large role in the progression and chemoresistance of cancer through its association with JAK/STAT signaling, biomarkers that are downstream of the NF-κB pathway [90]. Furthermore, a comprehensive multi-omics study that utilized single-cell transcriptomics and proteomics analyses identified 15 CAF gene subtypes that are associated with worse cancer prognosis, which is in large part associated with sustained NF-κB activation [91]. Likewise, gastric cancer cells overexpress the inhibin β subunit, which activates NF-κB signaling by secreting activin B and converting them into pro-tumorigenic CAFs, suggesting that targeting the NF-κB pathway will have significant effects on the TME and can serve as a new approach to cancer therapy [92].

## 7. Conclusions

The NF-κB pathway has been widely studied in terms of its effects on inflammation, immune responses, cell proliferation, survival, and apoptosis [93]. The purpose of this review is to highlight how the NF-κB pathway is also involved in cancer progression and metastasis and how further research into this pathway can provide insight into possible therapeutic strategies to target cancer progression and metastasis.

Current research has shown strong potential in targeting the NF-κB pathway when used with current therapies, such as chemotherapy, androgen deprivation therapy, and different herbal medicines [94]. This pathway was seen to help the most in cancers that form drug resistance with their current modalities, as inhibition of this pathway was effective in increasing sensitivity to the drug while reducing resistance. Research has shown moderate success in targeting the NF-κB pathway in hematologic malignancies; however, more research is needed on more complex and challenging-to-treat solid tumors such as prostate and colorectal cancer [17,18,19].

Furthermore, with advancements in research methods such as the use of organoids, patient-derived xenografts, and 3D culture modeling, and given that the tumor microenvironment is a complex ecosystem modulated by the NF-κB pathway, further research regarding therapeutics targeting the pathway should be explored in how the NF-κB pathway affects the TME and thus cancer initiation/progression [95].

Limitations include not knowing exactly how the NF-κB pathway affects other mechanisms within the human body, because the pathway is active throughout the body and is involved in other processes such as cardiometabolic processes and neuronal functioning [96,97]. The importance of the pathway in immune responses and tissue homeostasis suggests that inhibition of the pathway for cancer treatment may lead to unintended consequences with normal cellular function [98]. Using selective inhibitors that target specific subunits dysregulated in cancers should be explored. Additionally, biomarkers that can predict treatment response should continue to be explored. It has been noted in colorectal cancer that the p65 and p50 NFKB subunits have been shown to be biomarkers predictive of outcomes. Specifically, higher levels of p65 have been associated with more advanced tumor stage and lower survival, while p50 is a predictor of survival after radiotherapy in rectal cancer. The ability to enhance the cancer therapeutic effects while mitigating the effects on normal cellular function by targeting this pathway should be further explored to use the pathway as a therapeutic approach to improve outcomes for patients affected by cancer.

## Figures and Tables

**Table 1:** Key NF-κB Pathway Molecules in Different Cancer Types.

NF-κB pathway Molecules in Cancer Types	
Cancer Type	NF-κB Activation Pathway/Key Molecules
Breast Cancer	Wnt, TRIM32, RSL3
Colorectal Cancer	MUC13, EGFR
Lung Cancer	DDX24, TRIM32, CD146
Melanoma	P65, SPP1, IL-8, VEGF, BET, MCL1
Prostate Cancer	IκKβ, BCL-2
Gastric Cancer	P65, IL-8, Siva-1
Nasopharyngeal Cancer	STAT3, LMP1, miR-125b, BamHI-A
Bladder Cancer	Survivin, miR-29b, TRIM29

## Data Availability

No new data were created or analyzed in this study. Data sharing is not applicable to this article.

## Abbreviations

The following abbreviations are used in this manuscript:

5-FU	5-fluorouracil
ACE	Angiotensin-converting enzyme
AKBA	Acetyl-keto-beta boswellic acid
AS	Artesunate
Bic	Bicalutamide
BET	Bromodomain and extra-terminal domain
BXD	Banxia Xiexin Decoction
CAF	Cancer-associated fibroblasts
COX	Cyclooxygenase
CRPC	Castrate-resistant prostate cancer
DiHEP	Docosahexaenoic acid derivative
DMAPT	Dimethylamino parthenolide
EBV	Epstein–Barr Virus
EGFR	Epidermal growth factor receptor
ENZ	Enzalutamide
*H. pylori*	*Helicobacter pylori*
ICI	Immune checkpoint inhibitor
IKK	Inhibitory-kappa B kinase
IL-1	Interleukin-1
IL-1R	Interleukin-1 Receptor
LMP1	Latent Membrane Protein 1
LPS	Lipopolysaccharides
NF-κB	Nuclear factor kappa B
NPC	Nasopharyngeal carcinoma
NSCLC	Non-small cell lung cancer
PKC	Protein kinase C
PTX	Paclitaxel
SPP1	Secreted phosphoprotein 1
TME	Tumor microenvironment
TKI	Tyrosine kinase inhibitors
TLRs	Toll-like receptors
TRIM	Tripartite Motif
TNBC	Triple-negative breast cancer
TNF-a	Tumor necrosis factor-alpha
TNFR	Tumor necrosis factor receptors
TRAIL	TNF-related apoptosis-inducing ligand
UPP1	Uridine phosphorylase 1
